# Dental metal artifacts in magnetic resonance-based synthetic computed tomography for brain radiotherapy: Impact on dose, patient setup, and geometric distortion

**DOI:** 10.1016/j.phro.2026.100924

**Published:** 2026-02-13

**Authors:** Lisa Milan, Francesco Pupillo, Margherita Corsi, Matteo Coppotelli, Alessio Minoggio, Paula Sargenti, Stefano Moretto, Margherita Casiraghi, Maria Antonietta Piliero, Klaudia Krzekotowska, Davide Giovanni Bosetti, Gianfranco Pesce, Francesco Mosè Castronovo, Stefano Presilla, Thomas Zilli

**Affiliations:** a Medical Physics Division, Ente Ospedaliero Cantonale (EOC) - Imaging Institute of Southern Switzerland (IIMSI), Bellinzona, Switzerland; b Radiation Oncology, Ente Ospedaliero Cantonale (EOC) - Oncology Institute of Southern Switzerland (IOSI), Bellinzona, Switzerland; c Facoltà Di Scienze Biomediche, Università Della Svizzera Italiana (USI), Lugano, Switzerland; d Faculty of Medicine, University of Geneva, Geneva, Switzerland

**Keywords:** Synthetic CT, Brain MR-only, Metal dental implant, Dose comparison, Geometric distortion

## Abstract

**Background and purpose:**

Magnetic resonance (MR)-based synthetic computed tomography (sCT) can improve target and organs-of-interest delineation in brain radiotherapy. However, metallic implants may cause anatomical inconsistencies, dose inaccuracies, and distortions. This study evaluates the impact of dental metal artifacts on MR-only workflow.

**Materials and methods:**

Ninety-six patients underwent MR and CT for radiotherapy planning; fifty-two with dental implants undergoing standard or stereotactic treatments were eligible. Dose-volume metrics (D_mean_, D_2%_, and D_98%_) and γ-index analyses (1%/1 mm and 3%/3 mm) were used to compare dose for targets and organs. For a subgroup of 15 patients, further analyses were performed. Image quality of sCT was evaluated against planning CT using Mean Absolute Error (MAE) of Hounsfield units (HU) within targets and organs. Offline sCT-cone beam CT (CBCT) registrations were compared with online CT-CBCT matches. Sequences for B0-map, the map of main static magnetic field, quantified geometric distortion.

**Results:**

Median target dose differences remained within ±0.3%, with maximum of 3.7% for D_98%_ due to artifact-induced body contour changes. Median organ dose deviations were ±0.3%. Passing rates for γ-index were 97.8% and 100% for 1%/1 mm and 3%/3 mm, respectively. Average MAE was below 10 HU for brain, reaching 40 HU in bone-target regions. No differences were observed between CT-CBCT and sCT-CBCT registration. Geometric distortions remained within 1 mm, satisfying radiotherapy requirements.

**Conclusions:**

Despite dental metal artifacts, sCT demonstrate dose accuracy comparable to standard CT. The results support clinical use of sCT in brain radiotherapy, with caution when artifacts alter the body near targets.

## Highlights

• Dental metal implants cause artifacts on synthetic computed tomography.
• Similarity index shows no differences between synthetic and real computed tomography.
• Median dose differences were ±0.3%, except in regions adjacent to metal implants.
• Synthetic and real computed tomography provide equivalent positioning accuracy.
• Geometric distortion was <1 mm, supporting safe synthetic computed tomography use.

## Graphical abstract

**Figure:**
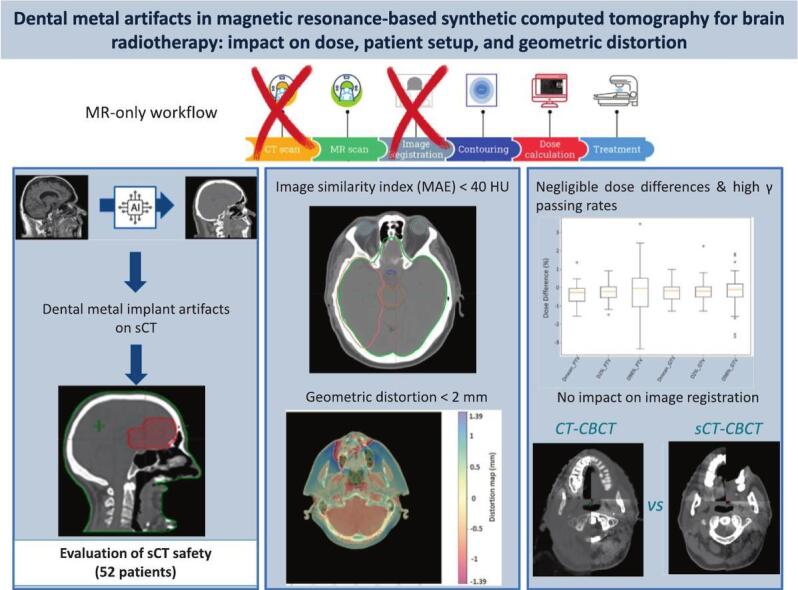

## 1 Introduction

Synthetic computed tomography (sCT) scans generated from magnetic resonance (MR) imaging have gained widespread acceptance in recent years, with several commercial tools approved for clinical use [1]. The MR-only workflow enables the creation of sCT from MR scans, providing electron density information for dose calculations without exposing the patient to ionizing radiation. Additionally, MR offers superior soft-tissue contrast, improving tumor and organs of interest delineation. By eliminating the need for image registration, the MR-only workflow has been shown to reduce target definition uncertainties [2]. Integrating MR data into treatment planning through sCT combines the strengths of both modalities and may improve treatment, particularly in brain cancer [2].

Despite these advantages, the use of sCT poses challenges, especially in the presence of intrabuccal metal implants. In fact, these can cause significant artifacts in both CT and MR images, impacting reconstruction and potentially affecting target contouring, dose calculations, and patient positioning. Metal-induced artifacts in MR include signal loss, failure of fat suppression, and geometric distortions [3]. These distortions are due to both scanner-specific factors, such as gradient non-linearity, imperfectly shimmed magnetic fields, and eddy currents, as well as patient-specific effects such as variations in magnetic susceptibility between metal implants and surrounding tissues [4]. This is especially critical in radiotherapy, where geometric distortions must remain below 1 mm near the lesion and under 2 mm over the treatment area [5]. To mitigate the effect of large metal implants, MR sequences should be designed to address this challenge by, for example, increasing the bandwidth and the matrix size, maintaining a good signal to noise ratio through additional excitations, and using spin echo instead of gradient echo when feasible. Other strategies include utilizing the short tau inversion recovery (STIR) for effective fat suppression, reducing echo spacing, minimizing water-fat shift, and using thinner slices to enhance image clarity and reduce metal-induced artifacts [3], [6].

Additionally, the algorithms used to generate sCT images from MR data are predominantly artificial intelligent (AI)-based, which introduces inherent variability in the resulting sCT that may not always align with the properties of standard CT scans [7]. These algorithms rely on complex deep learning models trained on large datasets to predict electron density values from MR imaging. While the AI-based models are highly accurate in many cases, they are influenced by several factors, such as the quality of the input MR data, the type of tissue being imaged, and the patient specific characteristics, including the presence of metal implants [8]. Given the widespread prevalence of dental metal implants, it is essential to understand their impact on sCT accuracy. While the impact of metal artifacts on CT-based dose calculations has been well documented [9], research on their influence on sCT images remains limited.

As the clinical use of sCT in the radiotherapy workflow continues to expand, rigorous evaluation of its performance in the presence of metal artifacts is essential to guarantee treatment safety and efficacy. This study evaluates the impact of dental metal implants on dose, patient positioning accuracy, and geometric distortion in patients with brain tumor lesions, aiming to determine the feasibility of sCT for routine clinical implementation.

## 2 Materials and methods

### 2.1 Patient cohort

Among the ninety-six consecutive patients who underwent both MR and CT acquisitions for radiotherapy planning, 12 (12.5%) were excluded due to bone-related artifacts, 20 (20.8%) had no imaging artifacts, five (5.2%) patients had tumors located outside the brain, and seven (7.3%) sCT were not available because of extensive artifacts preventing the reconstruction. The remaining 52 patients (54.2%), all with brain tumors and dental metal artifacts as the only source of imaging artifacts, were included in this retrospective study, comprising a total of 69 intracerebral planning target volumes (PTVs). Thirty-six lesions (52%) were treated with conventional or moderately hypofractionated radiotherapy for intracerebral gliomas or whole-brain palliative treatments, while thirty-three planning target volumes (PTVs) (48%) underwent stereotactic radiosurgery (SRS) for brain metastases. The median prescribed dose (interquartile range, IQ) for SRS treatments was 27 Gy (12–35 Gy) delivered in 1–5 fractions, with the prescription set at the 80% isodose line. For moderate hypofractionation, the median dose was 40.05 Gy (27.5–40.05 Gy), while for standard fractionation it was 60 Gy (47.7–60 Gy). All patients included in this study provided general informed consent for hospital-based research. The study contains no case details or other personally identifiable medical information.

For planning purposes, patients underwent a CT simulation scan on a Philips Brilliance Big Bore (Philips Healthcare, Best, The Netherlands) with tube voltage 120 kVp, slice thickness 1 mm and pixel sizes 1.1 mm x 1.1 mm. Immediately after, MR sequences were acquired using a 1.5 T Philips Ingenia® Ambition. Thermoplastic head immobilization was used during both CT and MR scans with three-point or double shell masks for conventionally fractionated or SRS treatments, respectively. Flexy, anterior, and embedded posterior coils were used for MR image acquisitions. A T1-weighted mDixon sequence was acquired in three-dimensional (3D) mode to generate the sCT using an AI-based algorithm, i.e. the MR for calculating attenuation (MRCAT) brain algorithm (version 4, Philips Oy, Vantaa, Finland). Additional sequences T1- and T2-weighted with contrast medium, and fluid-attenuated inversion recovery (FLAIR) were acquired for delineation of targets and organs of interest. The sCT images were generated automatically by the scanner simultaneously with the acquisition of other sequences. The reconstructed sCTs had 1 mm slice thickness and in-plane resolution of 0.68 mm × 0.68 mm.

Prior to analysis, sCT images were rigidly registered to the CT using the 6 degree-of-freedom image registration tool available in the Eclipse treatment planning system (TPS) (v. 16.1, Varian Medical Systems, Palo Alto, CA, USA). The relevant organs were delineated on CT images and transferred to the sCT with the exception of the body contour which was automatically generated on the sCT image, using the same threshold as CT (i.e. −350 Hounsfield unit (HU)).

### 2.2 Dose calculation

For each patient a volumetric-modulated arc therapy (VMAT) treatment plan was clinically optimized on CT images in the Eclipse TPS. Absorbed dose was calculated using the AcurosXB algorithm (AXB, version 16.1) employing a 1.25 mm grid size for SRS and 2.5 mm grid size for non-SRS treatments. To eliminate the influence of the CT calibration curve on the results, the calibration curve of our Philips Brilliance Big Bore scanner was used for converting HU into electron and mass densities for both the CT and sCT. Since the couch and immobilization devices are not visible on MR and therefore on sCT, and their inclusion would have required manual insertion with potential positioning inaccuracies relative to the planning CT (see Supplementary Materials, Fig. S1 and Table S1), these components were excluded from both sCT- and CT-based dose calculations [10].

The original treatment plan was recalculated on the sCT images, using identical plan parameters, maintaining the same number of monitor units. The dose-volume histogram (DVH) parameters, including D_2%_, D_98%_, and D_mean_, were assessed for both the PTVs and gross tumor volume (GTVs), as well as D_mean_ and/or D_2%_ of the optic chiasm, brainstem, lens, and eyes. The relative dose differences with respect to the CT plans (ΔD_Targets_) were evaluated for PTVs and GTVs, while for the organs, the dose differences (ΔD_organs_) were normalized to the prescribed dose [11].

(1) $$\Delta D_{\mathrm{Targets}}=\frac{D_{\mathrm{CT}}-D_{\mathrm{sCT}}}{D_{\mathrm{CT}}}$$

(2) $$\Delta D_{\mathrm{OARs}}=\frac{D_{\mathrm{CT}}-D_{\mathrm{sCT}}}{D_{\mathrm{prescribed}}}$$

Wilcoxon signed-rank test for paired test was used to compare metrics between planning CT and sCT. Global 3D gamma analysis was performed on the full dose grid (cut-off dose at 30%) at 3%-3 mm and 1%-1 mm, comparing the dose distributions on sCT and CT images for all patients.

### 2.3 Image similarities

The HUs of the sCT images were compared pixel-by-pixel (i) to the CT images using mean absolute error (MAE) as a similarity index, according to:

(3) $$\mathrm{MAE}=\frac{\sum_{i=1}^{N}\left|{\mathrm{CT}}_{i}-{\mathrm{sCT}}_{i}\right|}{N}$$

with N equal to the total number of pixel in the area of interest. The analyses were performed for the targets volumes (GTVs and PTVs), brain, bones, brainstem, optic chiasm, lens, and eyes on a subgroup of 15 patients representing the same distribution of artifact severity as observed in the entire population. The calculations were performed with Python code developed in-house.

### 2.4 Patient positioning accuracy

To assess patient positioning accuracy, CBCT images were retrospectively co-registered with sCT images for 15 patients (total of 139 registrations) and compared to CT-CBCT online treatments matching. The co-registration was performed by an experienced medical physicist using the 6-degree-of-freedom automatic registration tool in the Eclipse TPS, followed by a manual fine tuning.

The accuracy of the CBCT-CT and CBCT-sCT co-registrations was then evaluated by subtracting the registration matrix of the CBCT-sCT from that of the CBCT-CT, allowing for a comparison of the differences between the two. Wilcoxon signed-rank tests were performed for comparing the matching modalities.

### 2.5 Patient related geometric distortion

To estimate the patient related geometric distortion, the B0 map for each patient was evaluated voxel-by-voxel. An additional T1w 3D fast field echo (FFE) turbo sequence was acquired for a subgroup of 15 patients (see Supplementary Materials Table S2). The distortion B0 map was directly reconstructed at the MR console (Philips NeuroScience package for 1.5 T, Philips Oy, Vantaa, Finland): each pixel derives from the phase difference map corresponding to two images acquired with different echo times in the sequence. The images were then exported and converted through a Python script into millimeter absolute distortion (d) using the following equation [5], [12]:

(4) $$d=\frac{\Delta B_{0}}{BW}∙pixelsize\;$$

The geometric distortion was evaluated for the whole body, PTVs, bones, brain, nasal cavities, brainstem and optic chiasm.

## 3 Results

**Fig. 1:**
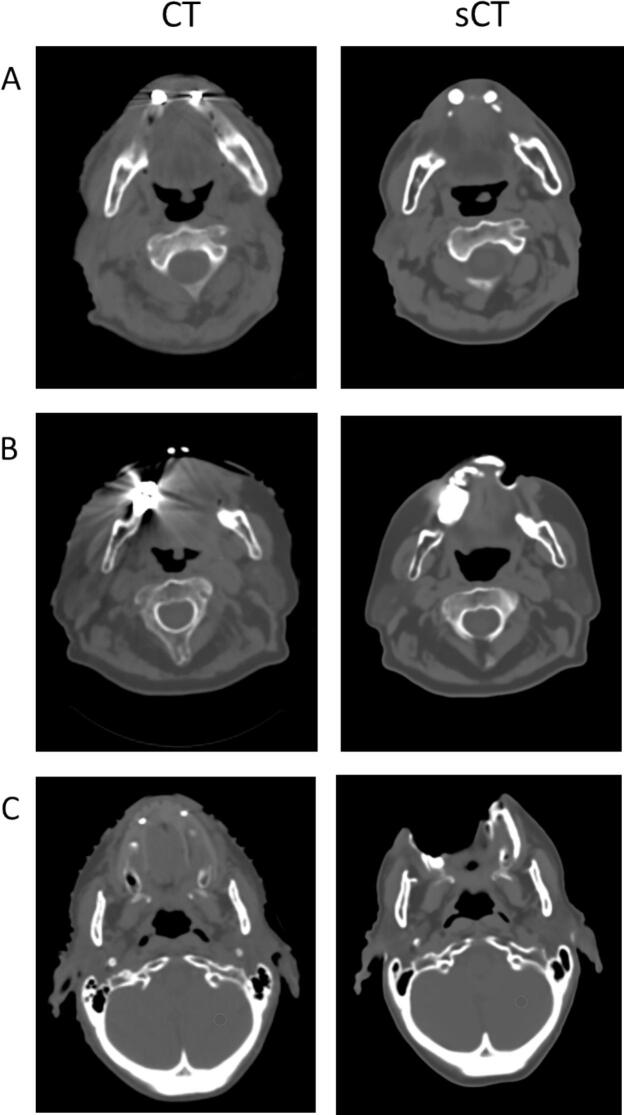
Qualitative examples of metal-induced artifacts in sCT images of patients with dental implants. (A) Case with minimal artifacts, comparable to the reference CT. (B) Case with more extensive distortions affecting the external body contour. (C) Critical case with significant signal loss near the lesion, potentially compromising anatomical accuracy. In our patient cohort, 27% of cases presented minimal artifacts with good overall image quality, 48% showed moderate artifacts with distortions of the external body contour, and in 25% of cases, severe artifacts such as signal voids (holes) in the body contour were observed.

Qualitative visual inspection of sCT originated from patients with dental implants revealed different levels of metal-induced artifacts (Fig. 1). In some cases (27%), artifacts were minimal and comparable to those observed in the reference CT images (Fig. 1-A). Conversely, 48% of cases exhibited more extensive distortions, changing the shape of the external body contour in the 25% of patients (Fig. 1-B). A particularly critical case involved a lesion adjacent to the artifact-affected area, where local signal loss overlapped with the anatomical region of interest (Fig. 1-C).

**Fig. 2:**
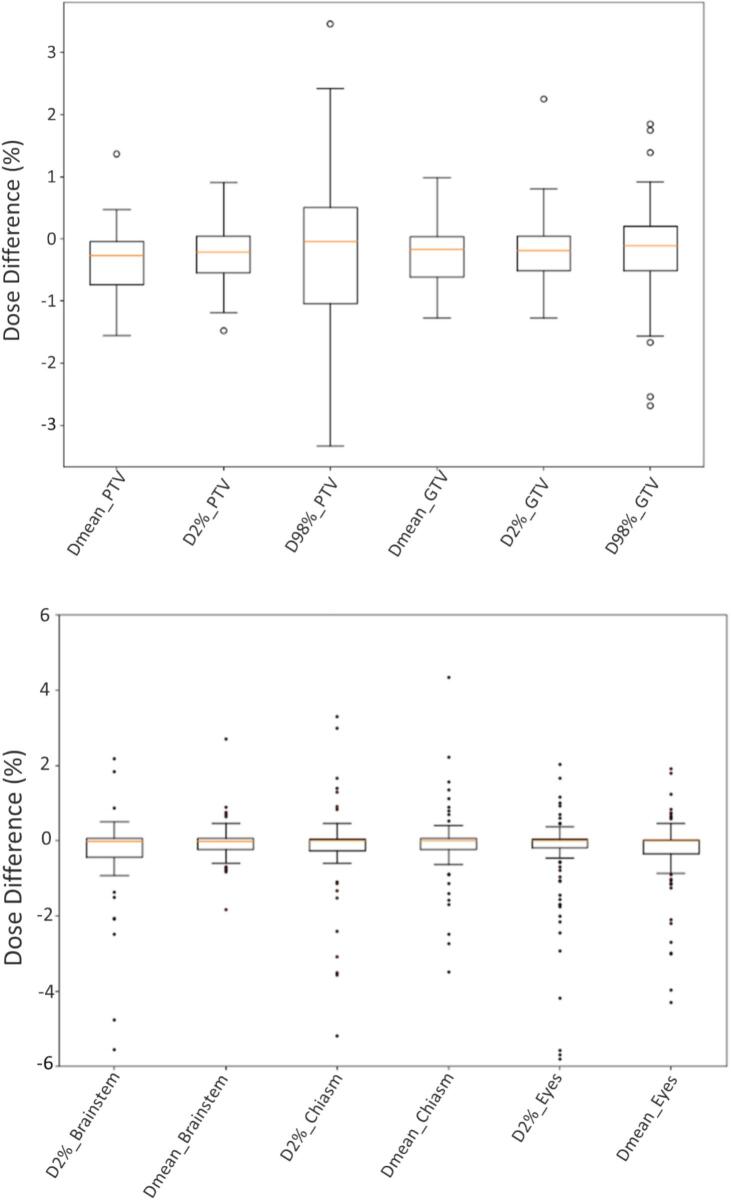
Dose comparison between CT- and sCT-based treatment plans for target volumes and organs of interest. Minor absolute dose differences (>2%) occurred in select cases where PTVs overlapped artifact-affected regions. Dose differences for the brainstem, optic chiasm, eyes, and lenses were also clinically non-significant though greater variability and outliers were noted in organs of interest.

Dose comparison between sCT- and CT-based treatment plans for target volumes and organs is presented in Table 1 and Fig. 2. For the PTV, the median D_mean_ was 31.9 Gy (inter-quartile range (IQR): 27.8–40.0 Gy) for CT-based plans and 32.0 Gy (IQR: 27.8–40.3 Gy) for sCT-based plans, with no statistically significant difference (p = 0.87). Similarly, no significant differences were observed in D_2%_ (34.4 Gy vs. 34.4 Gy, p = 0.85) or D_98%_ (30.0 Gy vs. 28.4 Gy, p = 0.56). Similar findings were observed for the GTV, with mean dose differing minimally between CT- and sCT-based plans (32.7 Gy vs. 32.9 Gy, p = 0.90), and both D_2%_ and D_98%_ showing close agreement (p = 0.84 and p = 0.74, respectively).

**Table 1:** Comparison of dose metrics between CT- and sCT-based treatment plans for target volumes and organs of interest. No statistically significant differences were observed (Wilcoxon’s test, all p > 0.05).

	Median [I-III IQR] CT dose [Gy]	Median [I-III IQR] sCT dose [Gy]	Wilcoxon’s test p-val
PTV D_mean_	31.9 [27.8–40.0]	32.0 [27.8–40.3]	0.87
PTV D_2%_	34.4 [30.8–42.1]	34.4 [30.8–42.2]	0.85
PTV D_98%_	30.0 [24.9–38.2]	28.4 [24.9–37.5]	0.56
GTV D_mean_	32.7 [29.3–40.9]	32.9 [29.6–40.9]	0.90
GTV D_2%_	34.7 [30.9–42.1]	34.5 [31.1–42.1]	0.84
GTV D_98%_	30.5 [26.9–39.6]	30.9 [27.3–39.4]	0.74
Brainstem D_mean_	1.9 [0.6–6.1]	1.9 [0.5–5.9]	0.99
Brainstem D_2%_	5.8 [2.8–19]	5.9 [2.8–19.2]	0.92
Optic Chiasm D_mean_	0.9 [0.2–7.8]	0.9 [0.2–7.5]	0.97
Optic Chiasm D_2%_	2.0 [0.3–15.1]	1.1 [0.2–9]	0.71
Eye Left D_mean_	0.6 [0.1–4.0]	0.6 [0.1–3.9]	0.98
Eye Right D_mean_	0.6 [0.1–4.0]	0.7 [0.1–4.0]	0.94
Lens Left D_mean_	0.4 [0.1–2.5]	0.4 [0.1–2.7]	0.78
Lens Right D_mean_	0.5 [0.1–2.4]	0.3 [0.1–2.7]	0.78

In a limited subset of cases (specifically for 5 PTVs), absolute dose differences exceeding 2% were noted for D_98%_, when the PTVs were aligned to artifact-affected regions with the beams intersecting these zones.

For the brainstem, both D_mean_ and D_2%_ showed negligible differences (p = 0.99 and p = 0.92, respectively). The optic chiasm, eyes, and lenses also exhibited statistically non-significant dose variations across all evaluated metrics (all p > 0.70). Notably, larger variation and more outliers were observed for organs compared to targets. Nevertheless, none of these differences were statistically significant (p > 0.05). Median gamma pass rates were 97.8% (IQR: 96.4–99.1%) for the 1%/1 mm criterion and 100% (IQR: 99.8–100%) for the 3%/3 mm criterion.

Quantitative image quality analysis (Table 2) revealed the largest HU discrepancies within bone structures, with a mean MAE of 146 HU ± 8.9. In contrast, soft tissues such as brain, and consequently the GTV, showed high agreement, with MAE values consistently below 15 HU. The higher MAE values in the PTV, by up to 49 HU compared with the GTV, are attributable to the inclusion of bone within the isotropic expansion margin used for PTV delineation.

**Table 2:** For a subgroup of 15 patients, the MAE ± standard deviation of HUs was evaluated for different anatomical structures. Highest variations occurred in bone (146 ± 8.9 HU), while soft tissues like brain and GTVs showed lower discrepancies (<15 HU). Elevated PTV variation (49 ± 35.5 HU) is due to bone inclusion in the GTV-PTV margins.

	Mean MAE [±SD] [HU]
GTVs	13.9 [±20]
PTVs	49 [±35.5]
Bones	146 [±8.9]
Brain	8.9 [±1.6]
Brainstem	6 [±1.9]
Optic Chiasm	23 [±21]
Lens	24 [±12.8]
Eyes	13.8 [±5.5]

**Fig. 3:**
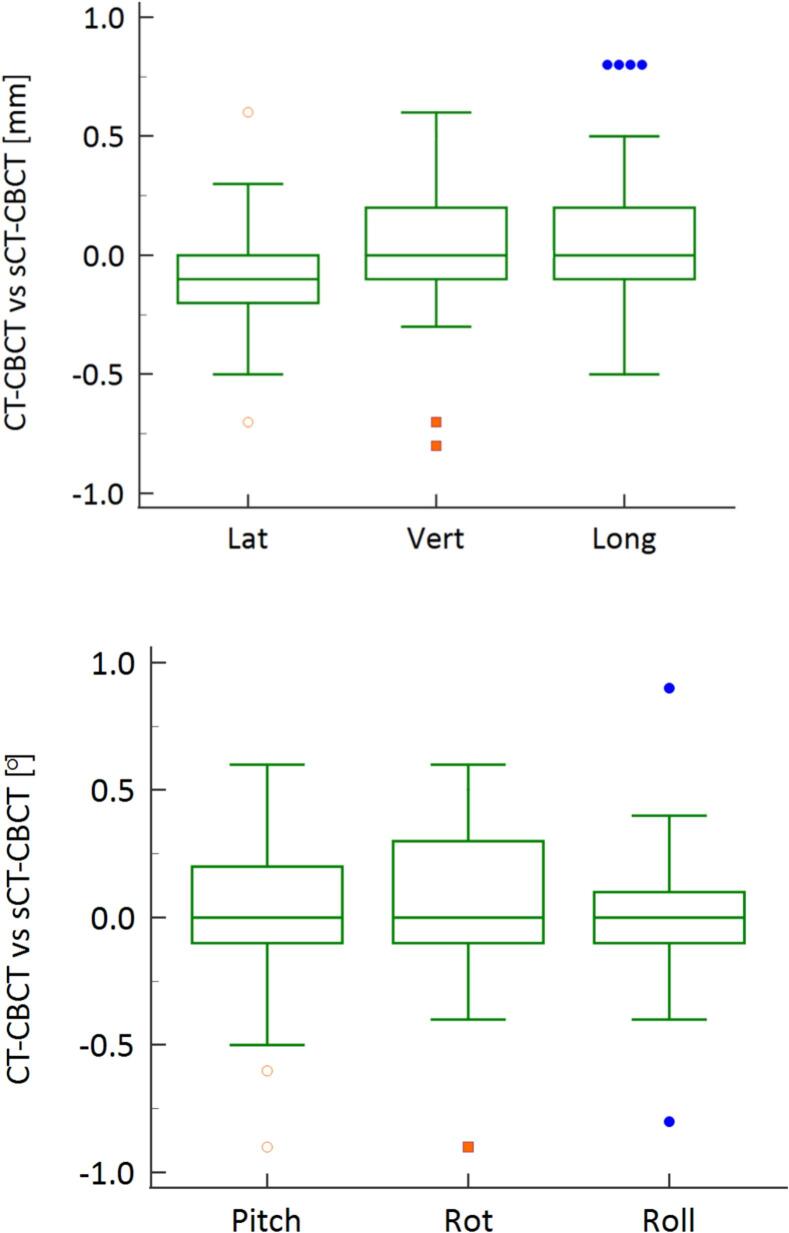
Registration accuracy of CT-to-CBCT vs. sCT-to-CBCT. Translational differences were minimal across lateral (0.17 ± 0.91 mm), vertical (–0.04 ± 0.78 mm), and longitudinal (0.13 ± 0.57 mm) axes. Rotational discrepancies were also low: pitch (–0.07° ± 0.4°), rotation (0.01° ± 0.21°), and roll (0.00° ± 0.16°). No statistically significant differences were found between the two registration methods (p > 0.05).

Inter-modality registration accuracy was evaluated by comparing translational and rotational differences between CT-to-CBCT and sCT-to-CBCT image registrations (Fig. 3). The mean ± standard deviation of translational discrepancies along the vertical, longitudinal, and lateral directions were 0.04 ± 0.78  mm, 0.13 ± 0.57  mm, and 0.17 ± 0.91  mm, respectively. Rotational discrepancies were 0.17° ± 0.4° (pitch), 0.01° ± 0.21° (rotation), and 0.00° ± 0.16° (roll). Statistical analysis confirmed no significant differences between the two registration methods across all axes (p > 0.05).

Analysis of patient-specific distortions (see Supplementary Materials Fig. S2) in the delineated targets and organs of interest remained within the sub-millimeter range (Table 3). For GTVs and PTVs, the median distortion values were 0.15 mm and 0.14 mm, respectively. Bony structures exhibited lower deviations, with a median value of 0.07 mm. In soft tissues, the brain showed a median deviation of 0.17 mm, while the nasal cavities presented slightly higher values, with a median of 0.24 mm. The brainstem demonstrated a systematic negative deviation, with a median shift of −0.26 mm. The maximum observed distortion was 1.46 mm, localized in the region of the nasal cavities.

**Table 3:** Geometric distortion values (median [interquartile range], mm) for anatomical structures. Distortions were generally sub-millimetric, with the largest deviations observed in the optic chiasm and nasal cavities.

	Median [I-III IQ] [mm]
GTVs	0.15 [0.00 – 0.21]
PTVs	0.14 [0.00 – 0.27]
Bones	0.07 [0.00 – 0.15]
Brain	0.17 [0.08 – 0.21]
Nasal cavities	0.24 [0.12 – 0.28]
Brainstem	−0.26 [-0.31 − −0.16]
Optic Chiasm	0.18 [0.1 – 0.44]

## 4 Discussion

This study assesses the impact of dental metal implants on the accuracy and reliability of MR-derived sCT images for radiotherapy planning. The results demonstrate that, despite challenges posed by metal artifacts, the generated sCT images are suitable for clinical use in treating brain lesions with either conventionally fractionated radiotherapy or SRS.

The DVH comparisons showed that the absorbed dose differences between CT and sCT treatment plans were minimal (within 0.5% for most patients) for both targets and organs. However, some clinically important deviations (up to a maximum of 3.7% for the D_98%_) were observed for targets in close proximity to dental implants. These deviations were particularly apparent when metal implant artifacts caused regions of air or resulted in the total loss of part of the jaw, leading to an underestimation of the dose in those areas. This suggests that when a metal implant is aligned to the target volume, a CT scan may be preferable to ensure accurate dose calculations and avoid potential under- or over-dosing of critical structures. In contrast, the larger range of relative dose differences observed for organs, compared to targets, can likely be attributed to two main factors: small variations in image registration and the specific locations of organs. Minor registration variations may cause inaccuracies in the transfer of contours between CT and sCT, particularly for smaller structures. Additionally, the outliers observed for these organs were located in low-dose regions and near air cavities. Consequently, the substantial relative dose differences were not considered clinically significant. These findings are consistent with prior studies that have demonstrated comparable levels of dose agreement between sCT images with and without artifacts [13], [14], [15]. Importantly, these differences were independent of the immobilization system, which was excluded from calculations, as the couch and devices are not visible on MR or sCT. Manual insertion would likely introduce positioning and dose inaccuracies, leading to larger variations than those caused by metal artifacts [10].

Comparisons of HUs show a high agreement between sCT and CT for soft tissues, with a mean MAE below 10 HU. However, the differences were more pronounced for bone structures, with mean discrepancies reaching up to 146 HU. When comparing the MAE for PTVs with that of GTVs, the MAE for PTVs was slightly higher due to the inclusion of bone tissue in the PTVs when created with an expansion from the GTVs in the proximity of the skull. These results are consistent with previous studies which reported similar MAE, indicating that the dental metal implants have a minimal impact on the HU assignment for both soft-tissues and bones [16], [17].

Regarding patient positioning, the minimal translational and rotational differences observed between CBCT-sCT and online CBCT-CT registrations remained within clinically acceptable thresholds, including for SRS treatments [12], [16], [18]. These findings indicate that sCT can be reliably used for patient positioning during radiotherapy, even in the presence of dental metal artifacts.

Geometric distortion in the MR images used for sCT generation was evaluated through B0 distortion maps, showing median distortions of less than 0.3 mm for all regions studied. As expected, larger distortions were observed in areas with air cavities, such as the sinuses [19], [20]. However, these distortions were not clinically significant, as values for targets and organs remained below the 1 mm threshold. Notably, the method used in our study to assess patient-specific distortions also accounted for B0 deviations within the MR system itself, leading to a slight overestimation of the patient-specific distortion.

This study has several limitations. Firstly, it is a retrospective analysis that excluded cases with severe artifacts preventing sCT reconstruction. Nevertheless, more than half of the reconstructed sCT images in our dataset (54.2%) contained dental metal artifacts, and approximately 7% could not be reconstructed at all due to these artifacts. In clinical practice, when sCT reconstruction fails, a planning CT scan must be acquired. Defining a precise threshold for the size or type of dental implant that causes sCT failure is challenging, as this depends on multiple factors such as the material composition, location, and extent of the implant. Accordingly, the present study specifically aimed to assess whether sCT images that were successfully reconstructed, despite significant metal artifacts, remained clinically usable. Finally, the use of a single vendor's sCT generation software may introduce bias associated with the underlying technology used, potentially limiting the generalizability of our finding to other systems.

In conclusion, to the best of our knowledge, this is the first study assessing the impact of dental metal implants on a commercially available brain sCT generation tool. Our results demonstrate that, even in the presence of such implants, sCT provides accurate and reliable data for dose calculation and patient positioning for brain radiotherapy. These findings align with existing literature and further support the feasibility of sCT-based planning and MR-only workflows in clinical practice.

## CRediT authorship contribution statement

**Lisa Milan:** Conceptualization, Methodology, Software, Investigation, Formal analysis, Writing – original draft. **Francesco Pupillo:** Investigation, Writing – original draft. **Margherita Corsi:** Investigation, Writing – review & editing. **Matteo Coppotelli:** Investigation, Writing – review & editing. **Alessio Minoggio:** Writing – review & editing. **Paula Sargenti:** Investigation, Writing – review & editing. **Stefano Moretto:** Writing – review & editing. **Margherita Casiraghi:** Writing – review & editing. **Maria Antonietta Piliero:** Writing – review & editing. **Klaudia Krzekotowska:** Writing – review & editing. **Davide Giovanni Bosetti:** Writing – review & editing. **Gianfranco Pesce:** Writing – review & editing. **Francesco Mosè Castronovo:** Writing – review & editing. **Stefano Presilla:** Writing – original draft, Project administration. **Thomas Zilli:** Writing – original draft, Supervision.

## Declaration of competing interest

The authors declare the following financial interests/personal relationships which may be considered as potential competing interests: The authors declare an ongoing collaboration with Philips; however, Philips had no involvement in the data collection, analysis, or preparation of this article.
