# Supplementary material for: Dental metal artifacts in magnetic resonance-based synthetic computed tomography for brain radiotherapy: Impact on dose, patient setup, and geometric distortion
Source: Phys Imaging Radiat Oncol. 2026 Feb 13;37:100924. doi: 10.1016/j.phro.2026.100924 (PMC12926589; doi:10.1016/j.phro.2026.100924)
Supplement: Supplementary Data 1 [file mmc1.docx]

**Supplementary materials**

Couch and immobilization devices are not visible on the MR images and hence on the sCT, therefore their inclusion would have required manual insertion. In practice, MR visible fiducials are placed in the mask pins and in the couch during MR acquisition to guide the registration of the support structures in the treatment planning system. This procedure introduce positioning inaccuracies compared to their actual location in the planning CT, leading to slight variations in the calculated dose up to 1.5% [1] unrelated to the presence of metal artifacts. Therefore, couch and immobilization systems were excluded from the CT-based dose calculation to ensure that any observed differences in dose distribution were exclusively attributable to the presence of metal artifacts. This was achieved by removing them from the body contour, as in the Eclipse TPS, only structures included within the body or explicitly defined as support structures are considered in the dose calculation.

In order to quantify these variations a sub-analysis on 5 patients showing the impact of errors in mask registration for both CT and sCT with and without masks and couch are reported in Table S2.


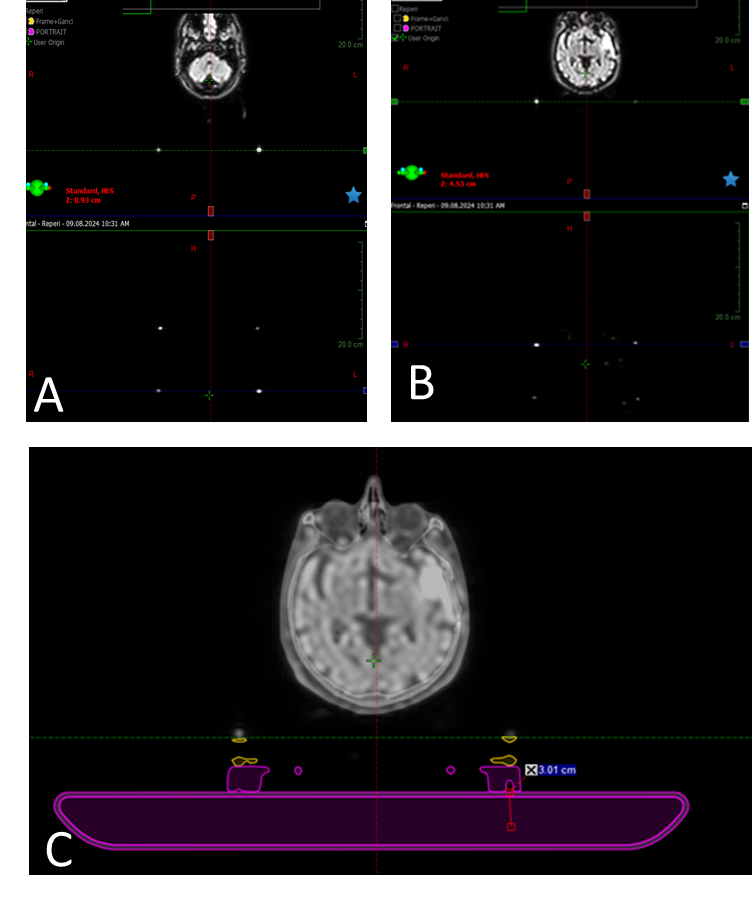


Figure S1: Visible fiducials placed in the couch (A) on the mask pins (B) during MR acquisition to guide the registration of the support structures in the treatment planning system (C).

Table S1: Targets perceptual differences in dose calculations with and without support structures for sCT and CT. The differences between CT and sCT are related to variations in how the immobilization devices are matched on the sCT.

|  | sCT with and without support structures | CT with and without support structures |
| --- | --- | --- |
|  | **Median [I-III IQR] [%]** | **Median [I-III IQR] [%]** |
| **PTV D_mean_** | -1.13% [-1.37%- -1.02%] | -1.74% [-2.29% - -1.47%] |
| **PTV D_2%_** | -1.18% [-1.55% - -0.95%] | -1.52% [-2.04% - -1.00%] |
| **PTV D_98%_** | -1.16% [-1.81% - -1.02%] | -1.83% [-2.32% - -1.55%] |
| **GTV D_mean_** | -1.30% [-1.51%- -1.18%] | -2.05% [-2.58% - -1.65%] |
| **GTV D_2%_** | -1.32% [-1.53% - -1.20%] | -1.95% [-2.58% - -1.60%] |
| **GTV D_98%_** | -1.08% [-1.46% - -0.93%] | -1.72% [-2.02% - -1.40%] |

*Table S2*: Imaging protocol for the sequence used for geometric distortion calculation.

|  | **B0 scan details** |
| --- | --- |
| **Scan mode** | T1 3D FFE TURBO |
| **Fold over direction** | RL |
| **Fat shift direction** | P |
| **Flip angle** | 60° |
| **Repetition time** | shortest |
| **Echo time** | In phase 4.6 ms |
| **NSA** | 2 |
| **Δ TE** | 3 ms |
| **Numebr of slices** | 62 |
| **FOV** | 238*238*2.5 |
| **Acq voxel size** | 3x3x2.5 |
| **Recon voxel size** | 1.2x1.2x2.5 |
| **Scan duration** | 4 mins |

**
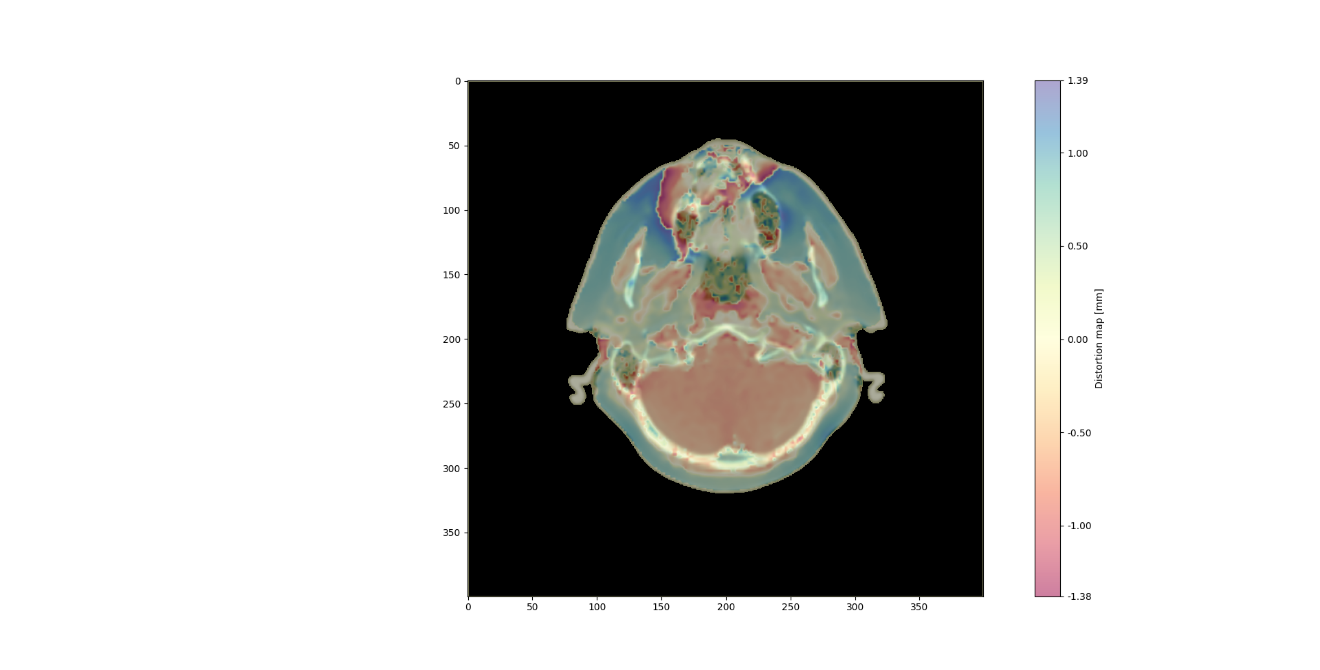
**

*Figure S2:* Example of a B0 map from a patient with a metallic implant. The image shows mild geometric distortion near the implant, corresponding to regions affected by magnetic field inhomogeneity. This distortion remained below 2 mm, which is the maximum geometric distortion allowed at the edge of the field of view for SRS treatment.

**Reference**

[1] Masitho, S., Grigo, J., Brandt, T., Lambrecht, U., Szkitsak, J., Weiss, A., Fietkau, R., Putz, F., Bert, C. (2023). Synthetic CTs for MRI-only brain RT treatment: integration of immobilization systems. Strahlenther Onkol, 199(8):739-748. https://doi.org/10.1007/s00066-023-02090-w
